# Supplementary material for: Digestibility of black soldier fly larvae (Hermetia illucens) fed to leopard geckos (Eublepharis macularius)
Source: PLoS One. 2020 May 11;15(5):e0232496. doi: 10.1371/journal.pone.0232496 (PMC7213736; doi:10.1371/journal.pone.0232496)
Supplement: Attachment — Submitted filename: Boykin et al..docx [file pone.0232496.s004.docx]

Overall this is a very well done experiment and provides valuable information to the veterinary and zoo community. The authors should be commended on their excellent work. That said I have some comments including a question if the data actually support the conclusion that “whole non-supplemented BSF larvae may not be a good source of calcium in leopard gecko”. I’ll address this issue below (comments 9 and to a lesser degree comment 11)

| Page | Line | Comment |
| --- | --- | --- |
| 2 | 29-30 & 35-36 | I’m not sure the data supports these statements. See comment #9 for more details why this might be. |
| 3 | 43-45 | While 3 years ago I would have agreed with this statement there is now data showing the calcium content of BSFL is highly variable (more so than I ever would have guessed) and diet dependent. A review of 3 articles (shown below) shows calcium levels from 0.12 (similar to that for other insects) to 6.66% (dry matter basis) and Ca:P ranging from 0.30:1 to 14.9:1. It would be good for the authors to mention this somewhere so veterinarians and zoo nutritionists understand that the diet plays a critical role in BSFL calcium content and that live soldier fly larvae sold commercially can varying widely in calcium content.  Spranghers T., Ottoboni M., Klootwijk C., Ovyn A., Deboosere S., De Meulenaer B., Michiels J., Eeckhout M., De Clercq P De Smet S., 2017. Nutritional composition of black soldier fly (*Hermetia illucens*) prepupae reared on different organic waste substrates. Journal of the Science of Food and Agriculture 97:2594-2600.  Tschirner M., and Simon A., 2015. Influence of different growing substrates and processing on the nutrient composition of black soldier fly larvae destined for animal feed. Journal of Insects as Food and Feed 1:249-259.  Wang S.Y., Wu L., Li B., Zhang, D. 2019. Reproductive potential and nutritional composition of *Hermetia illucens* (Diptera: Stratiomyidae) prepupae reared on different organic wastes. Journal of Economic Entomology doi: 10.1093/jee/toz296 |
| 4 | 65 | There is now data available to show that insects can be an excellent source of vitamin D if exposed to UV light which might help explain how nocturnal insectivores get their vitamin D. That article is Oonincx DGAB, van Keulen P, Finke MD, Baines FM, Vermeulen M, Bosch G. 2018. Evidence of vitamin D synthesis in insects exposed to UVb light. Nature - Scientific Reports DOI:10.1038/s41598-018-29232-w |
| 10 | 218-221 | It might be nice to tell the reader the breakout of the 10 geckos that lost weight and the 7 that were removed (i.e. how many came from each of the three groups) |
| 11 | 243-247 | I wonder if the authors might graph wt change by food intake (either % of body wt or perhaps ME) and they might be able to generate a nice regression line showing the maintenance energy requirements of these geckos. Might be a nice figure to add if there is a good correlation between intake and weight change. |
| 13 | Table 2 | Perhaps here or somewhere else it might be a good idea to show the readers the average nutrient composition of the BSFL used in this experiment. I realize the raw data is in the supplemental appendix but perhaps to help the reader the average data (at least moisture, protein, fat, ash, Ca and P) might be shown in the article. |
| 14 | Table 3 | Does the statistical significance change for the plasma vit A data if the sample that was reported as <20 is assigned a value of 0 rather than 20? Seems unusual to assign it a 20. |
| 14 | Table 3 | Does the statistical significance change for the liver vit A data if the outlier sample of 61.35 ug/g is included in the non-gut loaded group? |
| 17 | 338-343 | I think this section needs to be reworded since the data does not appear to be consistent with previous statements (see comment #1). The authors estimate that “when adjusted for digestibility the larvae supplied 9.2 g Ca/kg diet” which is 8-51% HIGHER than the estimated calcium requirement for growing leopard geckos that they cite (6.1-8.5 g Ca/kg diet). Add to this the fact that these geckos were adults likely means the calcium intake was likely 200-300% of the adult requirement. It is well known that calcium regulation in most species occurs in the intestine. To quote the NRC Mineral Tolerance of Domestic Animals (page 99) “During positive calcium balance intestinal mechanism for absorption are shut down in most species”. That would appear to be the case here as even with relatively low absorption the geckos were likely consuming 2-3 times their requirement. So while low digestibility of calcium in the exoskeleton is certainly a possible explanation realistically feedback mechanisms regulating calcium absorption likely play as much if not a bigger role in the low calcium digestibility observed here. |
| 17 | 344-345 | The statement “The increased phosphorus content would require a subsequent increase in calcium level to maintain the proper calcium to phosphorus level” seems overstated given how little data there is on optimum calcium-phosphorus ratios in reptiles and even for most vertebrates studied the ranges are fairly broad, typically 1:1 to 2:1. Suggest changing this to “The increased phosphorus content would require a subsequent increase in calcium level to maintain the SAME (emphasis mine) calcium to phosphorus level” |
| 17 | 356-359 | See the previous comment about calcium regulation (comment 9). As such a blanket recommendation regarding adding additional calcium to soldier fly larvae would seem to put animals at risk for excessive calcium intake which has been shown to inhibit the absorption of trace minerals putting the animal at risk for secondary trace mineral deficiencies. Add to that is the fact that the calcium content of black soldier fly larvae is highly variable and there is no standardized data for black soldier fly larvae sold commercially. As such I think this statement needs to either be removed or extensively modified. |
| 20 | 405-410 | Note Ploog reports plasma vitamin A values for Mississippi gopher frogs and while not a reptile it is an insectivore. Ploog C, Clunston R, Morris C, Iske C, Blanner W, Pessier A. 2015. Hypovitaminosis A: influence of three diets or topical treatment on hepatic, adipose, and plasma retinoid concentrations and presence of squamous metaplasia in Mississippi gopher frogs (*Rana capito servosa*). In Bissell H, Brooks M Eds. Proceedings of the Eleventh Conference on Zoo and Wildlife Nutrition, AZA Nutrition Advisory Group, Portland, |
| 20 | 442-443 | There is now data showing carnivores can convert beta-carotene to retinol although whether they can make sufficient retinol from beta-carotene to meet their needs is unclear (see Green, Tang, Lango, Klasing, & Fascetti. Domestic cats convert [2H8]-β-carotene to [2H4]-retinol following a single oral dose. J Anim Physiol Anim Nutr 96:681-92.) Since in some studies that show no conversion the animals were fed or were previously fed diets containing high levels of retinol (often from liver) it may be that feedback mechanisms designed to prevent vitamin A toxicity were in play. Note Mississippi gopher frogs appear to be able to convert carotenoids to retinol. See Ploog at al cited in the previous comment. |
| 22 | 483-485 | Agree however the amount of time required for depletion likely depends on vitamin A stores in the liver so depending on how the animals are fed it may take less than or much more than 6 months to show signs of deficiency. |
| 24 | 524-527 | I think these two sentences need to be modified based on the data shown and my comments (#9). |
